# The emerging molecular architecture of schizophrenia, polygenic risk scores and the clinical implications for gXe research

**DOI:** 10.1007/s00127-014-0961-6

**Published:** 2014-09-06

**Authors:** James B. Kirkbride

**Affiliations:** Sir Henry Dale Fellow, Division of Psychiatry, University College London, 2nd Floor Charles Bell House, 67–73 Riding House Street, London, W1W 7EJ UK

**Keywords:** Polygenic risk, Gene-environment interaction, Epidemiology, Schizophrenia, Psychiatric genetics

## Abstract

In this commentary I review the recent paper by Iyegbe et al. on "The emerging molecular architecture of schizophrenia, polygenic risk scores and the clinical implications for gXe research". I discuss how the paper advances our knowledge of polygenic risk scores for use, amongst others, in gene-environment interaction studies and the opportunities and challenges such approaches will bring to our understanding of the epidemiology of psychotic disorders, including schizophrenia.

The latest and largest genome-wide association train has just rolled through *Nature*, bringing with it the exciting news that 128 novel and established genetic loci for schizophrenia have been identified [1]. This news is rightly being heralded as a potentially major breakthrough in our understanding of genes important in the risk of schizophrenia, by both reproducing already established risk genes previously identified in subsamples of the same dataset, as well as identifying 83 previously unknown loci as potential aetiological and therapeutic targets [1].

Putting aside staple epidemiological concerns regarding heterogeneous control sampling of its more than 50 constituent samples, and possible selection biases inherent therein, the increased power to detect genes with small effect sizes brings welcome precision to the field of schizophrenia genetics, as it continues to disentangle the signal from the noise.

Such discoveries will also be a boon to researchers investigating the ways in which genetic and environmental influences combine to affect the risk of experiencing schizophrenia and other psychotic and genetically related psychiatric conditions [2]. One possible approach to gene–environment research is outlined by Iyegbe and colleagues [3] in their state-of-science review in a recent issue of *Social Psychiatry and Psychiatric Epidemiology*, in which they provide the background, rationale, challenges and methodological approaches to using polygenic risk scores in clinical and aetiological practice.

Iyegbe et al. [3] begin their article with a generally well-balanced and highly accessible overview of the historical development of the major genetic and environmental discoveries which have been elucidated in schizophrenia research. They are quick to recognise the redundancy in what should now be considered a sterile debate over “genetic” versus “environmental” causes. Given a myriad of genetic and environmental “loci” for schizophrenia dictate low specificity with regard to both exposure and outcome, it is natural to assume that mechanisms beyond main effects must influence the risk of a given psychiatric disorder, or that there are many mechanisms through which psychosis can occur at the individual level. Although discussed here in terms of gene–environment interactions [gXe], other possible mechanisms, including gene expression [4] and gene–environment correlation [5] require careful, equal consideration; both genetic and epidemiological expertise will be vital in developing longitudinal cohort studies capable of investigating such pathways. Such studies sit at the top of a relatively intuitive set of observational designs available in epidemiological research, which have various strengths and limitations depending on the exact research goal or question [6].

The authors [3] are particularly generous in their appraisal of findings in relation to “well-established” and “robust” socio-environmental risk factors associated with schizophrenia risk. Indeed in some cases, epidemiologists themselves may exercise more caution. For some risk factors, such as cannabis use [7] or very severe prenatal malnutrition [8], the evidence base is indeed strong. Elsewhere, however, while there is good epidemiological evidence that environmental factors such as migration [9] and ethnic minority status [10], or urban birth and upbringing [11, 12], are consistently associated with increased incidence, or risk, the exact social, biological or genetic exposures which these markers represent, remain unknown. Further research is required to carefully examine competing hypotheses, which include the influence of social stressors [13] such as discrimination [14, 15], inequality [16] and disadvantage [17], biological stressors [18] such as infection or malnutrition (including vitamin D) or effect modification via as yet untested candidate genes. Just as the large relative risks traditionally associated with a family history of psychosis may (in part) provide a summary measure of the totality of genetic (and shared environmental) risk now more accurately partitioned by genome wide association studies [GWAS], so might the risk associated with urban living or minority position be a summary marker for a range of deleterious environmental or genetic processes operating further along the causal pathway. For example, a recent study of 2.4 million people in Sweden suggests that selection processes (genetic or environmental) operating at the family level may be responsible for associations between deleterious environmental factors and later schizophrenia risk [19].

Indeed, the raison d’etre for any epidemiological study is not only to elucidate large relative risks but to explain variation in risk via careful measurement and scrutiny of other (traditionally, “confounding”) variables. Thus, as psychiatric genetics has traditionally subsumed the environment as a noise term in analyses, even the best epidemiological studies of psychotic disorder may have been limited in their ability to account for residual or unmeasured confounding, from both genetic and environmental factors, wherein the most valuable aetiological clues may lie. The “flaw” spotted by Iyegbe et al. [3] in one of our previous publications on the population impact of such factors and prevention of schizophrenia [20], thus arises from a more general limitation of our discipline; the challenge of conducting epidemiological studies of disorders of very rare incidence, using large representative and unbiased samples, with comprehensive measurement of all the social, biological, neurodevelopmental and genetic factors potentially influencing risk over the life course. As acknowledged in our original report [20] (pp. 7–8), we recognised “that other, unmeasured confounders may be important, including a family history of psychoses…[I]t is unlikely that psychosocial risk factors are often sufficient to cause psychosis, but rather interact with neurodevelopmental or genetic vulnerability.” Thus, we echo and support Iyegbe et al.’s [3] view (pp. 175) that there “is a relative paucity of datasets able to adequately assess the effect of joint exposure to genes and environment, which will be necessary for advancing aetiology and estimating the true proportion of disease which could be prevented by the removal of exposure to either or both sets of factors”.

It follows that risk prediction is a major focus of Iyegbe and colleagues’ review [3]. They eloquently outline the basis and method for the development of a polygenic risk score, drawing on available GWAS data. The polygenic score represents the within-person sum of all risk alleles for a given locus of interest multiplied by its (log) odds ratio as identified in a “training” sample of GWAS data. This polygenic score is subject to less sampling error as more data are added from GWAS consortia, but its psychometric properties currently still fall short of predictive validity necessary for use in the general population. More interestingly, however, there is increasing evidence that polygenic scores may be reaching threshold validity for predicting those at high risk of psychosis, which potentially yields exciting opportunities for early intervention research [21].

Iyegbe et al. [3] should be commended for writing a highly comprehensive review in clear, understandable language for epidemiologists and other psychiatric researchers not working regularly with complex GWAS data. In doing so their paper raises a number of questions about the use of polygenic risk scores which could further aid both research, and the non-familiar researcher. One useful next stage, for example, would presumably be to identify the social, clinical, developmental and environmental correlates of high polygenic scores in ultra-high risk samples and test how these then map on to later transition to the development of first episode psychosis. Careful research is needed to determine whether polygenic risk scores can be augmented with information on family history of disorder for the practical identification of at-risk groups, or in order to test putative gXe interactions in schizophrenia. Researchers will need convincing that a polygenic risk score for these purposes can enhance detection of gXe effects over and above those provided by a family history of psychosis, which may be cheaper, easier and more practical to implement in large observational or experimental research designs as a marker for high genetic risk. Iyegbe et al. [3] discuss the potential role of polygenic scores in gXe studies, including under Genome Wide Environment Interaction Studies (GWEIS) approaches. Space and care are devoted to the complexities and controversies involved in detecting gXe interactions (see also [22, 23]), including sample sizes needed to demonstrate their presence, and more problematically in terms of power, their absence.

A further challenge to advocates of a polygenic risk approach in gXe studies is to explain how it can enhance the understanding of the molecular architecture of schizophrenia, and the pathways through which polygenic risk might combine with environmental factors to influence the likelihood of schizophrenia. Since there may be several different pathways through which various combinations of genetic and environmental factors act, synergistically or directly, it is unclear how a single polygenic score would help clarify those genes or brain systems involved. One question which arose from Iyegbe et al.’s [3] stimulating review was the extent to which it would be possible to identify “latent” polygenic risk scores which sought to cluster genetic risk according to different theoretical pathways of disease causation (dopaminergic, glutamatergic, GABAergic, calcium channels, etc.). Given theoretical and empirical evidence to suggest that social environmental stressors may act most strongly on genes involved in dopamine sensitization [18, 24], such an approach would presumably increase the a priori theoretical justification and statistical power to detect putative gXe interactions and the mechanisms implicated therein. I commend Iyegbe et al. [3] on a highly readable review of a complex area and hope that this builds on contemporary gXe endeavours [2] to lay the rails for future discourse, collaboration and discovery between the social and genetic sciences.
